# Association between psychosocial safety climate and depression risk among Korean workers

**DOI:** 10.4178/epih.e2025044

**Published:** 2025-08-13

**Authors:** Soo Kyung Cho, Seong-Sik Cho, Maureen F. Dollard, May Young Loh, Mo-Yeol Kang

**Affiliations:** 1 Department of Public Health, Graduate School, The Catholic University of Korea, Seoul, Korea; 2 Department of Occupational and Environmental Medicine, College of Medicine, Dong-A University, Busan, Korea; 3 Psychosocial Safety Climate Global Observatory, Centre for Workplace Excellence, Justice & Society, University of South Australia, Adelaide, Australia; 4 Department of Occupational and Environmental Medicine, Seoul St. Mary’s Hospital, College of Medicine, The Catholic University of Korea, Seoul, Korea

**Keywords:** Psychosocial safety climate, Depression, Workplace, Mental health, Korea

## Abstract

**OBJECTIVES:**

Psychosocial safety climate (PSC) reflects an organization’s commitment to safeguarding workers’ psychological health and safety. While international evidence links low PSC to poor mental health outcomes, its association with depression has not been well established in Korea. This study aimed to examine the relationship between PSC and depression among Korean workers, utilizing a large-scale, population-based survey.

**METHODS:**

We analyzed data from 5,337 wage employees who participated in the fifth wave of the Korean Work, Sleep, and Health Study. Depression was measured using the 9-item Patient Health Questionnaire, and PSC was assessed with a validated 4-item scale. Participants were classified into low-risk, intermediate-risk, and high-risk groups based on their PSC scores. Multivariable logistic regression was conducted to estimate the association between PSC and depression, with stratified analyses performed according to gender, age, and occupational characteristics.

**RESULTS:**

The prevalence of depression increased as PSC scores decreased. Compared to the low-risk group, the intermediate-risk and high-risk PSC groups exhibited 1.19 times and 2.69 times higher risks of depression, respectively, suggesting a clear exposure-response relationship. Stratified analyses indicated that associations were stronger among individuals without union representation or access to occupational health and safety resources.

**CONCLUSIONS:**

These findings underscore the critical role of PSC in workplace mental health. Promoting a high PSC may help reduce depression risk and support mental well-being among workers. Interventions considering vulnerable subgroups are warranted to create psychologically safer work environments in Korea.

## GRAPHICAL ABSTRACT

[Fig f1-epih-47-e2025044]

## Key Message

• This study demonstrates that lower psychosocial safety climate (PSC) scores are associated with a significantly higher risk of depression among Korean workers.

• The risk of depression was 1.19 times higher in the intermediate-risk PSC group and 2.69 times higher in the high-risk PSC group compared with the low-risk group.

• Findings highlight the importance of strengthening PSC in workplaces to improve employee mental health and guide national policies.

## INTRODUCTION

Depression is a prevalent mental disorder, affecting approximately 5% of adults worldwide, and is recognized as a leading cause of disability globally [1-4]. In the United States, the economic burden of major depressive disorder among adults rose from US$236 billion in 2010 to US$326 billion in 2018, with workplace costs constituting a significant proportion of this increase [5]. Depression and anxiety disorders have been linked to the loss of approximately 12 billion working days each year, resulting in costs to the global economy exceeding US$1 trillion annually [6]. These statistics illustrate the substantial economic and social impact of depression on individuals, communities, and organizations. Therefore, addressing depression is essential not only for improving individual well-being but also for enhancing societal productivity and economic stability.

Korea has persistently reported the highest suicide rate among Organization for Economic Cooperation and Development [7], highlighting the urgent need to address mental health issues nationwide. In Korea, a demanding work culture characterized by long working hours and significant performance pressure has been associated with negative mental health outcomes. For example, 61% of compensated suicides between 2016 and 2017 were found to be related to overwork, including factors such as long working hours and increased workload [8].

Psychosocial safety climate (PSC) refers to an organization’s prioritization of policies, procedures, and practices designed to protect employees’ psychological health and safety [9]. PSC reflects senior management’s commitment to addressing work-related stressors and creating a supportive environment that prioritizes employee well-being over productivity. As a leading indicator of workplace psychosocial risk, PSC shapes job design—including job demands and available resources—and influences social-relational aspects of work, such as relationships between supervisors and employees and among colleagues. High PSC levels are linked to reduced job strain, greater resource accessibility, and improved psychological health outcomes. In contrast, low-PSC environments are reported to contribute to increased stress, reduced well-being, and a higher risk of depression, emphasizing the role of PSC as a foundational precursor for establishing healthy and sustainable workplaces.

Although there is growing recognition of the role of psychosocial factors in shaping workplace mental health [10], research specifically examining the relationship between PSC and depression remains limited, particularly in Korea, where studies on PSC are scarce and its impact on employee well-being has yet to be thoroughly investigated [11]. Addressing this gap in the literature requires research evaluating how PSC affects depression risk within Korean workplaces. Additionally, examining this relationship in the general working population can yield valuable insights into the cultural and organizational contexts that may influence the PSC-depression link, thereby informing both global and region-specific strategies for workplace mental health.

Therefore, in this study, we examined the association between PSC levels and depression risk, incorporating stratified analyses by gender, age, occupational characteristics, and other socio-demographic variables. By considering various factors, the objective of this study was to provide a comprehensive understanding of how PSC affects mental health across different workforce groups. These findings may inform actionable recommendations for both organizational and policy-level interventions aimed at improving worker well-being and mental health resilience.

## MATERIAS AND METHODS

### Study participants

This study utilized data from the fifth wave of the Korean Work, Sleep, and Health Study, an ongoing nationwide panel study initiated in 2022 [12]. Participants were recruited in August 2024, through the EMBRAIN online survey platform. All participants provided voluntary informed consent prior to completing the survey. The sample was stratified by gender and age and included individuals from a wide range of occupational backgrounds. Among those who met the initial eligibility criteria (wage earners aged 19 years or older), 5,337 respondents (2,805 men and 2,532 women) provided complete responses to the survey.

### Measurements

#### PSC

A validated 4-item version of the PSC questionnaire was used to assess workers’ perceptions of the PSC in their workplace [13]. The scale encompasses 4 domains, each rated on a 5-point Likert scale ranging from 1 (strongly disagree) to 5 (strongly agree). The items assessed: (1) senior management’s support for stress prevention through involvement and commitment; (2) participation and consultation in occupational health and safety issues with employees, unions, and occupational health and safety representatives; (3) the extent to which contributions to resolving occupational health and safety concerns are listened to within the organization; and (4) the degree to which stress prevention is implemented across all layers of the organization. Total PSC scores range from 4 to 20, with higher scores reflecting a more positive PSC. Scores were categorized as follows: >12 indicating low-risk, 9-12 indicating moderate-risk, and <9 indicating high-risk. These cutoff values were based on benchmarks established by Berthelsen et al. [14], which were originally developed in the context of occupational health and safety (OHS) practices rather than depression. More recent benchmarks derived from the PSC-12, and aligned with depressive symptomatology, suggest alternative thresholds: a PSC-12 score of ≤37 (equivalent to 12.33 on the PSC-4) indicates high-risk, and ≤26 (equivalent to 8.66 on the PSC-4) indicates very high-risk [15]. Applying these newer thresholds suggests that risk classification may differ depending on the benchmark used, but the overall categorization remains unchanged.

#### Risk of depression

Depression was measured using the Patient Health Questionnaire-9 (PHQ-9), a widely used self-report screening instrument for assessing the severity of depressive symptoms in population-based studies. The Korean-translated version of the PHQ-9 has demonstrated high reliability and is frequently used in research [16,17]. The PHQ-9 consists of 9 items corresponding to the Diagnostic and Statistical Manual of Mental Disorders, Fourth Edition criteria for major depressive disorder, with responses ranging from 0 (not at all) to 3 (nearly every day). An example item is: “During the past 2 weeks, how often were you bothered by feeling down, depressed, or hopeless?” Total scores range from 0 to 27, with higher scores indicating greater symptom severity. A score of 10 or above is generally considered to represent moderate to severe depression, and this cutoff demonstrates good sensitivity (88%) and specificity (88%) for major depressive disorder in primary care settings [18].

#### Covariates

Demographic and work-related variables included gender, age, education level, occupation, workplace size (number of workers), employment contract, working time contract, labor union, and OHS availability. Gender was categorized as men or women, and age was divided into age groups of 20-30 years, 40-49 years, 50-59 years, and 60-69 years. Educational attainment was classified as middle or high school, college, university, or postgraduate. Occupations were categorized based on the 7th Korean Standard Classification of Occupations as follows: white-collar (managers, professionals, and office workers); pink-collar (service and sales workers); and blue-collar (workers in agriculture, forestry, fishery, crafts, machine operation, assembly-line work, and manual labor). Workplace size was divided into 3 groups: <50, 50-300, and >300 employees. Working hours were categorized as full-time or part-time. Additionally, data on the presence of labor unions and the availability of OHS resources were collected.

### Statistical analysis

Descriptive statistics were used to summarize mean PSC-4 scores and PSC risk levels across demographic and work-related characteristics. Differences in the distribution of PSC risk levels (categorized as low, moderate, or high) according to demographic and occupational characteristics were assessed using Pearson’s chi-squared (χ²) test. Logistic regression models were employed to examine the association between PSC and depression risk. The dependent variable was the presence of major depressive symptoms (coded as yes=1, no=0), and the independent variable was PSC risk level. Three logistic regression models were constructed: a crude model with no adjustments; a model adjusted for age and gender; and a fully adjusted model that further controlled for occupation, educational level, workplace size, and employment contract. The low-risk PSC group served as the reference category, and odds ratios (ORs) with 95% confidence intervals (CIs) were calculated for the moderate-risk and high-risk PSC groups. Stratified logistic regression analyses were conducted to examine whether the association between PSC and depression differed according to demographic and occupational subgroups. Analyses were stratified by gender, age group, educational attainment, occupation type, workplace size, employment contract, working time, labor union presence, and OHS availability. To evaluate effect modification, interaction terms were included in the logistic regression models, and the statistical significance of each interaction was assessed using likelihood ratio tests. All statistical analyses were performed using R version 4.5.0 (R Foundation for Statistical Computing, Vienna, Austria), with statistical significance set at p-value <0.05.

### Ethics statement

All participants provided signed informed consent, and confidentiality and anonymity were maintained throughout the study. The study was approved by the Institutional Review Board of Dong-A University of Korea (IRB No. 2-1040709-AB-N-01-202202-HR-017-06).

## RESULTS

Table 1 presents the characteristics of the 5,337 participants. The mean PSC score was 9.51 (standard deviation [SD], 3.82). Regarding PSC risk levels, 18.8% of participants were classified as low-risk, 39.2% as moderate-risk, and 41.9% as high-risk. Men were more likely than women to be in the low-risk category (21.8 vs. 15.6%) and less likely to be in the high-risk category (37.0 vs. 47.5%). The proportion of individuals in the high-risk group decreased with age, from 47.8% among those aged 20-39 years to 29.3% among those aged 60-69 years. Pink-collar workers reported the highest mean±SD PSC scores (9.69±3.81). Workers employed in larger workplaces (>300 employees) and those with access to OHS resources exhibited higher PSC scores than their counterparts.

Logistic regression analyses demonstrated that higher PSC risk levels were significantly associated with increased risk of depression. Compared to the low-risk group, the adjusted odds ratio (aOR) for depression was 1.20 (95% CI, 1.01 to 1.42) in the moderate-risk group and 2.69 (95% CI, 2.22 to 3.26) in the high-risk group, after adjusting for age, gender, occupation, education level, workplace size, and employment contract (Table 2). These results confirm the overall trend that lower PSC scores (reflecting higher risk) are associated with increased depression risk.

Notably, the increase in depression risk was greater for women than for men, suggesting that poor PSC has a more pronounced impact on women. Across age groups, depression risk increased in the low-PSC group for all ages, with the most marked increase observed in the 20-39-year age group. Individuals with graduate-level education were also more likely to experience a higher risk of depression when PSC was poor. As PSC scores declined, the risk of depression increased across all occupational categories, with the most substantial increase observed in workplaces with fewer than 50 employees. Full-time workers exhibited a greater increase in depression risk than part-time workers as PSC scores decreased. The risk of depression also rose in groups without labor union membership when PSC scores were lower. This increase was particularly significant among those without access to OHS services (Table 3).

When formal interaction terms were incorporated into the model, only the interaction between PSC risk levels and OHS availability reached statistical significance (p=0.044), indicating a potential effect modification. No statistically significant interactions were found for gender, age, education level, occupation, workplace size, working time contract, or union status.

## DISCUSSION

In the present study, we identified PSC as a significant social determinant of depression risk, with poor PSC associated with a substantial increase in the likelihood of depressive symptoms in an exposure-response manner: the intermediate-risk and high-risk groups demonstrated 1.19-fold and 2.69-fold higher risks of depression, respectively, compared to the low-risk group. Stratified analyses indicated that this association was especially pronounced among those without access to OHS resources. These findings suggest the critical role of PSC in protecting mental health across diverse segments of the workforce.

The findings of this study are consistent with those of previous research, showing that PSC is a critical precursor to organizational psychological safety and employee mental health [10]. For example, a prospective cohort study of 1,084 full-time Australian employees examined the relationship between PSC and the onset of major depressive symptoms over a 12-month period [19]. The authors found that employees working in environments with low PSC were 3 times more likely to develop new major depressive symptoms compared to those working in high-PSC settings, even after adjusting for potential confounders. Dormann et al. [20] addressed challenges in interpreting cross-lagged panel model (CLPM) results using incidence rates and risk ratios, which are more common metrics in epidemiological research. Drawing on longitudinal data from 1,905 employees in the Australian Workplace Barometer Project, they applied continuous-time modeling and Monte Carlo simulations over 4 years, translating CLPM estimates into incidence rates and risk ratios. Their findings revealed that individuals with poor PSC experienced over 100% more depression incidents than expected over the subsequent 4 years. A recent scoping review synthesizing 93 studies found consistent evidence that higher PSC is associated with lower job demands, better access to resources, reduced workplace mistreatment, and improved psychological outcomes, including reduced distress, burnout, and depression [21]. In line with these findings, our results indicate that high PSC likely mitigates the risk of depression.

Stratified analyses further suggested that the detrimental effects of low PSC may vary across employee demographics, providing both anticipated and exploratory insights. However, these findings should be interpreted with caution, as formal interaction tests did not show statistical significance except for 1 subgroup. A significant effect was observed among workers lacking access to OHS resources, where the aOR reached 3.86. This finding emphasizes the importance of organizational support systems in counteracting the negative impacts of low PSC [22]. Access to OHS resources emerges as a pivotal factor for safeguarding employees’ mental health [23], particularly in low-PSC environments. Together, these results indicate the necessity for organizations to cultivate a robust PSC, employing targeted interventions to address the specific needs of vulnerable employee groups. By strengthening PSC, organizations can proactively reduce depression risk and promote overall employee well-being.

Several limitations of this study should be noted. First, the cross-sectional design precludes the establishment of causality between PSC and depression risk, as data were collected at a single point in time, preventing temporal assessment. Cross-sectional data also limit the ability to evaluate workers’ previous mental health status. Longitudinal or intervention-based research is needed to clarify the directionality and persistence of these associations. Second, reliance on self-reported data may introduce biases such as social desirability bias, where participants respond in ways they perceive as favorable, and recall bias, which can result in inaccuracies in reporting past experiences [24,25]. Although both the PSC-4 and PHQ-9 are validated instruments and have strong psychometric properties, their subjective nature remains a limitation. Such biases may have affected the validity of our findings. Third, while the PHQ-9 is a validated and widely used screening tool, it does not offer a clinical diagnosis of depression. The lack of clinical interviews or diagnostic confirmation could have resulted in misclassification. Furthermore, the study did not account for participants’ use of antidepressant medications, which might have influenced symptom severity or reporting. Fourth, although PSC is conceptualized as a shared, organizational-level climate, our analysis treated it as an individual-level perceptions due to the absence of workplace or departmental identifiers in the dataset. This may underestimate the true influence of collective PSC and does not account for potential clustering effects among employees within the same organization. Future research should use multilevel modeling approaches that explicitly account for workplace clustering, better reflecting the group-level nature of PSC and distinguishing between individual and organizational-level influences on mental health outcomes. Finally, the use of random sampling may have affected the representativeness of the study population, potentially limiting the generalizability of the results. Investigating across diverse cultural and industrial contexts could enhance the applicability of these findings and guide targeted interventions.

In conclusion, this study identified low PSC as a significant factor associated with increased depression risk, with variables such as access to organizational resources potentially moderating this association. These findings have important implications for workplace health policies and national mental health strategies. Organizations should consider incorporating PSC assessment into routine psychosocial risk management, in alignment with emerging global standards. Senior management should implement organizational interventions—including leadership training, integration of PSC into OHS systems, and targeted support for vulnerable groups—to foster a psychologically safe and supportive work environment. At the national level, governments need to consider developing PSC assessment standards and guidelines, promoting PSC across industries and occupations. Such strategies are especially relevant in Korea, where workplace mental health continues to be a pressing societal concern.

## Figures and Tables

**Figure f1-epih-47-e2025044:**
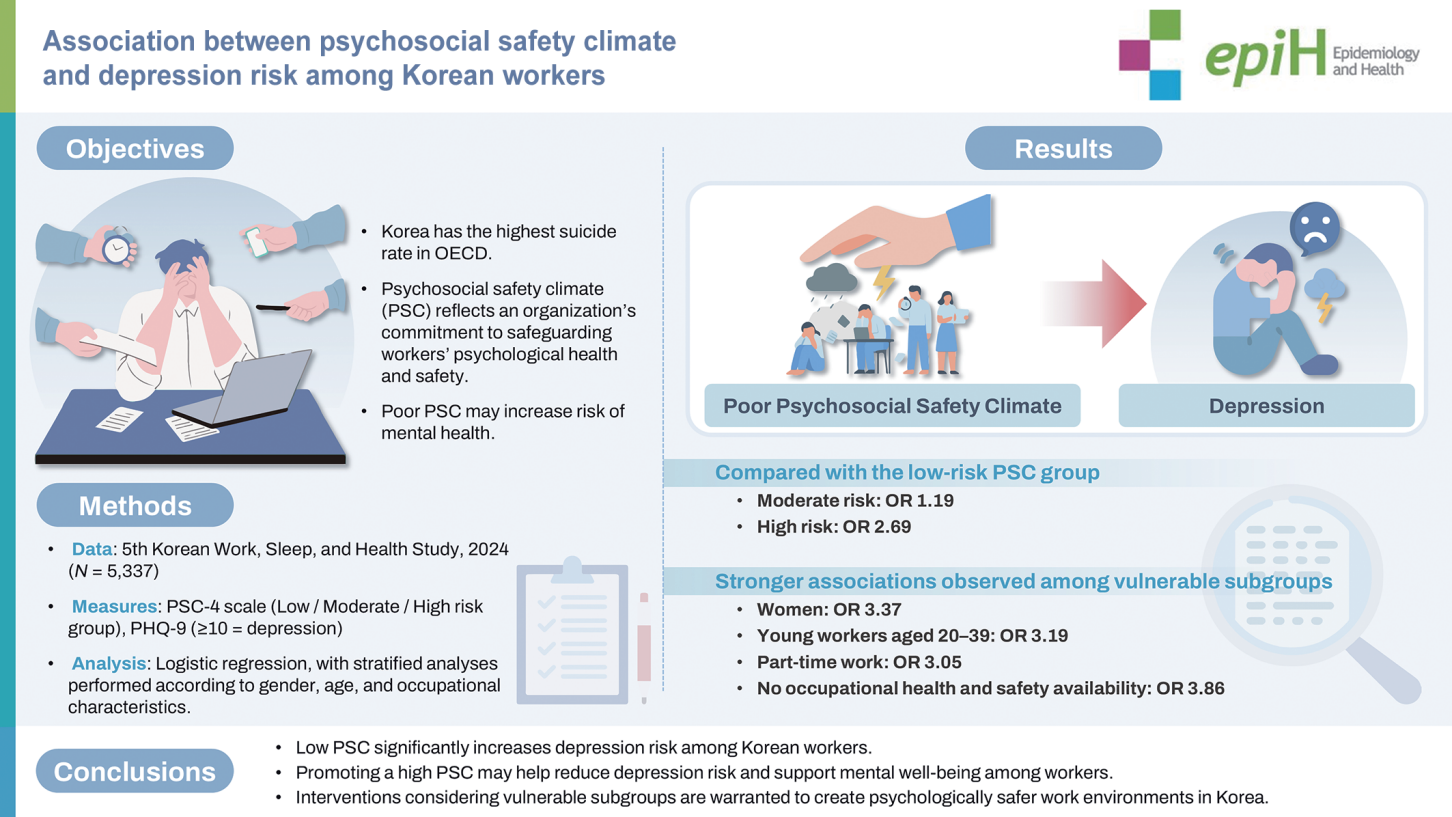


**Table 1.** Mean PSC-4 score and PSC risk level according to demographic variables and work characteristics

Variables	n	PSC-4 score	PSC risk level			p-value^1^
			Low	Moderate	High	
Overall	5,337	9.51±3.82	1,006 (18.8)	2,093 (39.2)	2,238 (41.9)	
Gender						<0.001
Men	2,805	9.98±3.76	612 (21.8)	1,157 (41.2)	1,036 (37.0)	
Women	2,532	8.98±3.82	394 (15.6)	936 (37.0)	1,202 (47.5)	
Age (yr)						<0.001
20-39	2,075	9.06±3.97	365 (17.6)	719 (34.7)	991 (47.8)	
40-49	1,285	9.16±3.76	205 (16.0)	500 (38.9)	580 (45.1)	
50-59	1,264	10.01±3.64	264 (20.9)	542 (42.9)	458 (36.2)	
60-69	713	10.51±3.51	172 (24.1)	332 (46.6)	209 (29.3)	
Education level						<0.001
Middle or high school	676	9.39±3.72	102 (15.1)	294 (43.5)	280 (41.4)	
College	935	9.16±3.91	157 (16.8)	349 (37.3)	429 (45.9)	
University	3,109	9.50±3.82	603 (19.4)	1,195 (38.4)	1,311 (42.2)	
Graduate school	617	10.15±3.72	144 (23.3)	255 (41.3)	218 (35.4)	
Occupation						0.195
White-collar	4,083	9.48±3.83	780 (19.1)	1,572 (38.5)	1,731(42.4)	
Pink-collar	429	9.69±3.81	80 (18.6)	188 (43.8)	161 (37.5)	
Blue-collar	825	9.51±3.78	146 (17.7)	333 (40.4)	346 (41.9)	
Workplace size (no. of workers)						<0.001
<50	2,317	9.04±3.76	353 (15.2)	907 (39.1)	1,057(45.6)	
50-299	1,508	9.38±3.80	266 (17.6)	578 (38.3)	664 (44.0)	
≥300	1,512	10.33±3.79	387 (25.6)	608 (40.2)	517 (34.2)	
Working time contract						<0.001
Full-time	5,046	9.46±3.82	943 (18.7)	1,957 (38.8)	2,146 (42.5)	
Part-time	291	10.28±3.79	63 (21.6)	136 (46.7)	92 (31.6)	
Labor union						<0.001
Yes	2,187	10.56±3.76	604 (27.6)	896 (41.0)	687 (31.4)	
No	3,150	8.77±3.68	402 (12.8)	1,197 (38.0)	1,551 (49.2)	
OHS availability						<0.001
Yes	2,908	10.47±3.72	757 (26.0)	1,205 (41.4)	946 (32.5)	
No	1,677	8.22±3.64	179 (10.7)	580 (34.6)	918 (54.7)	
Don’t know	752	8.64±3.52	70 (9.3)	308 (41.0)	374 (49.7)	

Values are presented as mean±standard deviation or number (%). PSC, psychosocial safety climate; OHS, occupational health and safety.

1 From the Pearson chi-square test.

**Table 2.** Depression risk according to psychosocial safety climate levels^1^

Risk level	Model 1	Model 2	Model 3
Low-risk	1.00 (reference)	1.00 (reference)	1.00 (reference)
Moderate-risk	1.22 (1.03, 1.45)	1.20 (1.01, 1.43)	1.19 (1.00, 1.41)
High-risk	3.10 (2.58, 3.74)	2.73 (2.26, 3.31)	2.69 (2.22, 3.26)

Values are presented as odds ratio (95% confidence interval).

1 Model 1: Crude (unadjusted); Model 2: Age and gender adjusted; Model 3: Age, gender, occupation, education level, workplace size, and employment contract–adjusted.

**Table 3.** Logistic regression analysis of depression risk across PSC groups by each variable

Variables	PSC risk level			p for interaction
	Low	Moderate	High	
Gender				0.140
Men	1.00 (reference)	1.05 (0.84, 1.31)	2.37 (1.85, 3.05)	
Women	1.00 (reference)	1.49 (1.12, 1.97)	3.37 (2.73, 4.59)	
Age (yr)				0.716
20-39	1.00 (reference)	1.20 (0.87, 1.65)	3.19 (2.24, 4.21)	
40-49	1.00 (reference)	1.09 (0.78, 1.68)	2.20 (1.44, 3.34)	
50-59	1.00 (reference)	1.20 (0.86, 1.66)	2.35 (1.62, 3.40)	
60-69	1.00 (reference)	1.18 (0.80, 1.74)	3.09 (1.95, 4.94)	
Education level				0.469
Middle or high school	1.00 (reference)	0.86 (0.47, 1.55)	1.87 (0.96, 3.59)	
College	1.00 (reference)	1.43 (0.90, 2.26)	3.50 (2.12, 5.82)	
University	1.00 (reference)	1.18 (0.93, 1.49)	2.58 (1.99, 3.33)	
Graduate school	1.00 (reference)	1.17 (0.68, 1.99)	3.90 (2.08, 7.48)	
Occupation				0.938
White-collar	1.00 (reference)	1.24 (1.02, 1.51)	2.76 (2.21, 3.45)	
Pink-collar	1.00 (reference)	0.95 (0.47, 1.85)	1.68 (0.77, 3.64)	
Blue-collar	1.00 (reference)	1.16 (0.75, 1.77)	2.88 (1.81, 4.61)	
Workplace size (no. of workers)				0.368
<50	1.00 (reference)	1.12 (0.84, 1.49)	2.82 (2.06, 3.86)	
50-299	1.00 (reference)	1.19 (0.85, 1.64)	2.75 (1.92, 3.93)	
≥300	1.00 (reference)	1.26 (0.93, 1.70)	2.43 (1.72, 3.46)	
Working time contract				0.870
Full-time	1.00 (reference)	1.17 (0.98, 1.40)	2.66 (2.18, 3.25)	
Part-time	1.00 (reference)	1.38 (0.67, 2.78)	3.05 (1.33, 7.21)	
Labor union				0.930
Yes	1.00 (reference)	1.19 (0.92, 1.54)	2.79 (2.12, 3.66)	
No	1.00 (reference)	1.20 (0.95, 1.53)	2.57 (1.92, 3.47)	
OHS availability				0.044
Yes	1.00 (reference)	1.08 (0.88, 1.32)	2.21 (1.73, 2.82)	
No	1.00 (reference)	1.78 (1.21, 2.61)	3.86 (2.59, 5.73)	
Don't know	1.00 (reference)	0.66 (0.32, 1.29)	1.86 (0.86, 3.82)	

Values are presented as odds ratio (95% confidence interval). PSC, psychosocial safety climate; OHS, occupational health and safety.
